# Application of causal forests to randomised controlled trial data to identify heterogeneous treatment effects: a case study

**DOI:** 10.1186/s12874-025-02489-2

**Published:** 2025-02-22

**Authors:** Eleanor Van Vogt, Anthony C. Gordon, Karla Diaz-Ordaz, Suzie Cro

**Affiliations:** 1 Imperial College London, London, UK; 2 University College London, London, UK

**Keywords:** Causal machine learning, Subgroup analysis, Critical care, Treatment effect heterogeneity, Causal inference, Randomised controlled trial

## Abstract

**Background:**

Classical approaches to subgroup analysis in randomised controlled trials (RCTs) to identify heterogeneous treatment effects (HTEs) involve testing the interaction between each pre-specified possible treatment effect modifier and the treatment effect. However, individual significant interactions may not always yield clinically actionable subgroups, particularly for continuous covariates. Non-parametric causal machine learning approaches are flexible alternatives for estimating HTEs across many possible treatment effect modifiers in a single analysis.

**Methods:**

We conducted a secondary analysis of the VANISH RCT, which compared the early use of vasopressin with norepinephrine on renal failure-free survival for patients with septic shock at 28 days. We used classical (separate tests for interaction with Bonferroni correction), data-adaptive (hierarchical lasso regression), and non-parametric causal machine learning (causal forest) methods to analyse HTEs for the primary outcome of being alive at 28 days. Causal forests comprise honest causal trees, which use sample splitting to determine tree splits and estimate treatment effects separately. The modal initial (root) splits of the causal forest were extracted, and the mean value was used as a threshold to partition the population into subgroups with different treatment effects.

**Results:**

All three models found evidence of HTE with serum potassium levels. Univariable logistic regression OR 0.435 (95%CI [0.270, 0.683]. *p* = 0.0004), hierarchical lasso logistic regression standardised OR: 0.604 (95% CI 0.259, 0.701), lambda = 0.0049. Hierarchical lasso kept the interaction between the treatment and serum potassium, sodium level, minimum temperature, platelet count and presence of ischemic heart disease. The causal forest approach found some evidence of HTE (*p* = 0.124). When extracting root splits, the modal split was on serum potassium (mean applied threshold of 4.68 mmol/L). When dividing the patient population into subgroups based on the mean initial root threshold, risk differences in being alive at 28 days were 0.069 (95%CI [-0.032, 0.169]) and − 0.257 (95%CI [-0.368, -0.146]) with serum potassium ≤ 4.68 and > 4.68 respectively.

**Conclusions:**

The causal forest agreed with the data-adaptive and classical method of subgroup analysis in identifying HTE by serum potassium. Whilst classical and data-adaptive methods may identify sources of HTE, they do not immediately suggest subgroup splits which are clinically actionable. The extraction of root splits in causal forests is a novel approach to obtaining data-derived subgroups, to be further investigated.

**Supplementary Information:**

The online version contains supplementary material available at 10.1186/s12874-025-02489-2.

## Background

To identify beneficial new treatments, randomised controlled trials (RCTs) focus on estimating the average treatment effect (ATE) of an intervention. The ATE can be thought of as the causal contrast between the mean potential outcomes of the treated and untreated [1]. This captures the typical treatment response across the entire trial population. However, there may be patients who had a much better or worse response than the average; that is, individual treatment effects can vary significantly from the ATE [2].

Critical care is a particularly high-stakes arena with resource allocation constraints [3] and a heterogenous population [4]. Intensive care unit (ICU) treatments are costly in terms of time, money, and person-time [5]. Diseases such as sepsis [6], community-acquired pneumonia [7] and acute respiratory distress syndrome [8] have many causes and aetiologies. Prompt, effective treatment is crucial for patients with such conditions in ICUs to improve survival [9]. However, many recent trials in this arena have concluded a null ATE [10, 11]. A null average result could lead to the end of research into an intervention. If heterogeneous treatment effects (HTEs) are better understood, research into an intervention could continue in patients likely to benefit, thus reducing research waste. Fair and optimal allocation of resources must be based on robust evidence [3]. Identifying who responds best to treatments and making this information accessible to clinicians responsible for treatment decisions is paramount to improving patient care.

Classical methods that exist for estimating heterogeneous treatment effects (HTEs) for subgroups require pre-planned subgroup analyses and a large sample size to be adequately powered [12]. Typically, the interaction between a single pre-specified patient characteristic and the treatment effect is tested individually [13]. Testing multiple characteristics in the same dataset can lead to a large risk of making a type I error [14].

The area of causal machine learning (CML) focuses on estimating ATEs and HTEs. There are a variety of methods for estimating these. For ATEs, targeted maximum likelihood (TMLE) [15] and double machine learning [16] are becoming increasingly common. For additionally estimating HTEs, causal forests (CFs) [17], and R-learners [18] are novel proposals gaining popularity. The CF approach is a tree-based method for estimating conditional average treatment effects (CATE), that is, the ATE as a function of a set of patient characteristics [19]. There are some examples of CF being implemented in social science research settings [17], but only a few applications to clinical trial settings [20]. The novelty and lack of exploration of these methods warrant this exploration and comparison with classical approaches to subgroup analysis.

Once the CATE is estimated, it may be of interest to generate subgroups with significantly different ATEs. Methods such as hierarchical clustering [21] and latent class analysis (LCA) [22] have been used to split patient populations into subgroups with differing treatment effects. An issue that arises from these methods is that there is no control for the fact that subgroups have been learnt using the sample at hand, which may lead to false discoveries. To ensure correct inference, the causal forest approach uses a recommended “honest” approach where the data is split into two: one subset is used to determine the splits in the trees, and the other subset is used to estimate the CATE, which we expand on further in the methods section. The causal forest uses variable importance to unpick which variables drive treatment effect heterogeneity in the trial sample. Further, an “omnibus test” can be applied to the resulting CATEs to determine the presence of heterogeneity [23].

CFs can yield complexly defined subgroups, as they are a combination of trees (with leaves defined by interactions). What defines these subgroups may not be immediately interpretable, making them difficult to communicate to clinicians and patients. For the CF, we propose a novel approach to separate the patient population into more clinically actionable subgroups based on the most common initial splits made by trees in the CF [24].

This article aims to explore the causal forest approach for estimating HTEs and compare it to i) the classical approach of conducting univariable interaction tests and (ii) data-adaptive methods utilising the hierarchical lasso (as an intermediate step). Throughout, we consider the utility of the methods for generating hypotheses for future research. Exploration is conducted through reanalysis of a critical care RCT, the VANISH trial [24].

## Methods

### Trial Summary

We used data from the VANISH trial, which was a 2 × 2 factorial trial including 408 randomised patients which compared early vasopressin vs. norepinephrine (standard care) and corticosteroids vs. placebo for patients with septic shock being treated in ICUs in England. The primary outcome used in the trial was the number of days alive and free from kidney failure in the 28 days after randomisation, a composite outcome of 28-day mortality and days free of kidney injury. This paper focuses on modelling the treatment effect for the binary outcome of 28-day mortality for the first-line treatments, early vasopressin versus norepinephrine. The trial did not detect a significant difference in 28-day mortality between arms (30.9% in the vasopressin group versus 27.5% in the norepinephrine group, risk difference: 3.4% 95%CI [-5.4–12.3%]). This original result is dependent on the underlying logistic regression model being correctly specified and the assumption that the treatment effect is homogeneous. If this is not the case, this null result may have been due to between-participant differences. If the homogeneity assumption is violated, the confidence intervals suggest that some patients experienced positive treatment effects and some negative. Therefore, it is of interest to explore the HTEs and identify the patients who may have benefitted from, or may have been harmed by, the treatment.

### Data handling

The VANISH trial collected a variety of patient baseline variables, daily measurements, and outcomes at day 28. All baseline measurements with missingness < 30% were considered possible treatment effect modifiers. These include demographic information and components of ICU APACHE II and SOFA illness severity scores (physiological measurements such as arterial pressure and temperature) and are listed in the supplementary material. Categorical variables were one-hot encoded (each category is represented with a binary indicator variable), and continuous variables were centred and scaled to have a mean of 0 and a standard deviation of 1.

### Classical approach– univariable regression

The classical approach of conducting subgroup analysis through testing each covariate-treatment interaction in a separate logistic regression model was initially used. For each of the $\:k=1,\dots\:,K$ potential covariate modifiers, where $\:{X}_{k}$ denotes the $\:{k}^{th}$covariate of interest considered as an effect modifier, the following model was fit in each instance:

$$\:logit\left(\text{{\rm\:E}}\left[Y|W,{\varvec{X}}_{\varvec{k}}\right]\right)\:=\:{\beta\:}_{1,k}W+{{\upbeta\:}}_{2,k}{X}_{k}+{{\upbeta\:}}_{3,k}W*{X}_{k},$$

where $\:Y$ is the mortality binary outcome and $\:W$ is the binary treatment indicator. In this situation, we are interested in the coefficients $\:{\beta\:}_{3,k}$ for all $\:{X}_{k}$. This model assumes a linear relationship between each of the variable $\:{X}_{k}$ and the outcome on the logit scale.

To account for the increase in type I error, which builds up when multiple tests are performed, we conservatively applied the Bonferroni multiple testing correction, that is, 0.05 divided by the number of tests being carried out. Results of the univariable analysis are visualised using a volcano plot, which plots effect sizes on the x-axis and $\:{\text{log}}_{10}\left\{p\right\}$-values on the y-axis.

### Machine learning approach– hierarchical Lasso

As an intermediary step between the classical method and the most up-to-date causal machine learning methods, we employed a variable selection technique to select which baseline covariates had strong interactions with the treatment. Hierarchical lasso penalisation (based on group lasso) [25] was applied to a logistic regression model, including the treatment, all baseline characteristics and all interaction terms between the treatment and baseline variables $\:{X}_{k}$, k = 1,…K.

$$\:logit\left(\text{{\rm\:E}}\left[Y|W,\:\varvec{X}\right]\right)\:=\:{\beta\:}_{1}W+\:\sum\:_{k\:=\:1}^{K}\left\{{\beta\:}_{2,k}{X}_{k}+\:{\beta\:}_{3,k}W*{X}_{k}\right\}.$$

The penalisation imposes a strong hierarchy such that

$$\:{\beta\:}_{3,k}\:\ne\:\:0\:\Rightarrow\:\:{\beta\:}_{2,k}\:\ne\:\:0\:and\:{\beta\:}_{1}\:\ne\:0.$$

This meant that if a covariate by treatment interaction was kept in the model, the main effect term for the variable in the interaction and treatment was also retained. The penalty term was selected using 5-fold cross-validation. This method was implemented using the “glinternet” package in R [25]. When using centred and scaled independent variables in a logistic regression, the magnitude of the log odds ratios are interpretable as variable importance measures. We refer to these as standardised odds ratios in the results section. In this work, we used this to understand the importance of the features retained by the hierarchical lasso.

Variable selection models, such as the hierarchical lasso, do not typically generate uncertainty estimates for adjusted coefficients in their selected models. Estimating uncertainty using data that informed model selection may lead to overly optimistic estimates. To demonstrate the uncertainty in estimates obtained through the hierarchical lasso, we employ a one-step selective inference procedure for the lasso proposed by Lie et al. [26]. However, we highlight that the confidence intervals presented for lasso estimates are not to be interpreted causally.

### Causal machine learning

The primary estimand of interest for causal machine learning methods is the so-called Conditional average treatment effect, which can be written in terms of potential outcomes for the outcome Y under the two possible treatments denoted $\:W\in\:\left\{\text{0,1}\right\}$ as,

$$\:\tau\:\left(x\right)=E\left[Y\left(1\right)-Y\left(0\right)|\:X=x\right],$$

Where $\:X\:=\:x$ is the baseline covariate vector. Under consistency, no interference and unconfoundedness (which holds by design in this case, as we are in a randomised setting), the CATE is $\:\tau\:\left(x\right)=E\left[Y\left|W=1,\:X=x\right]-E\left[Y\right|W=0,\:X=x\right].$

### Causal forest (CF)

The causal forest (CF) is a non-parametric CML method which estimates the CATE [19]. The causal forest begins by assuming that in a small neighbourhood $\:N\left(x\right)$ (which will be defined by the splits) the CATE $\:\tau\:\left(x\right)$ is constant. Assuming a partially linear model $\:E\left[Y|W,X\right]=m\left(X\right)+\:W\tau\:\left(x\right)\:$, we can estimate $\:\tau\:\left(x\right)$ through the individuals $\:i$ in the neighbourhood of $\:x$, denoted by $\:i:{X}_{i}\in\:N\left(x\right)$

$$\:\widehat{\tau\:}\left(x\right)=\:\frac{\sum\:_{\{i:{X}_{i}ϵ\:N(x\left)\right\}}^{}\left\{\left({{W}_{i}-\widehat{p}\left({X}_{i}\right)\left)\right(Y}_{i}-\widehat{m}\left({X}_{i}\right)\right)\right\}}{{\sum\:}_{\{i:{X}_{i}ϵN(x\left)\:\right\}}^{}{\left\{{W}_{i}-\widehat{p}\left({X}_{i}\right)\right\}}^{2}}$$

Where $\:i$ indexes the individuals in the data. The propensity score $\:p\left(X\right)=E\left[W|\:X\right]$ is obtained by regressing W on X many times non-parametrically via random forest, and treatment-free mean outcome $\:m\left(X\right)=E\left[Y|X\right]$ is obtained by regressing Y on X, not including W, via random forests. The estimate of $\:m\left(X\right)$ can be re-written as:

$$\eqalign{\hat m\left( x \right) & = \>\sum {\>_{i = 1}^n} \alpha {\>_i}\left( x \right){Y_i},\> \cr{\rm{where}}\>\alpha {\>_i} \left( x \right) &= \>{1 \over B}\sum {\>_{b = 1}^B} {{1\left( {\left\{ {{X_i} \in \>\>{L_b}\left( x \right),\>i\> \in \>{{\cal S}_b}} \right\}} \right)} \over {\left| {\{ i:{X_i} \in \>{L_b},\>i\> \in \>{{\cal S}_b}} \right|}}, \cr} $$

where $\:{\alpha\:}_{i}\left(x\right)$ are weights that represent the frequency at which the $\:i$th observation falls into the leaf containing $\:x$. B is the number of trees in the causal forest, $\:{L}_{b}\left(x\right)$ represents the leaf in tree b containing observation$x$and $\:{\mathcal{S}}_{b}$denotes the set of trees which use the observation $i$.

Out-of-bag observations, those not used in the training of the model, are used to generate the “residuals” $\:Y-\widehat{E}\left[Y|X\right]\:$and $\:W-\widehat{E}\left[W|X\right].\:$Then, the data is split using “causal trees”, which, instead of choosing splits to minimise prediction error, greedily search over covariate thresholds to maximise the difference in the ATE between two leaves resulting from a split so that the estimated treatment effects are similar within the leaf (approximating homogenous treatment effects within a leaf), while they maximise the difference between the resulting leaves, (capturing heterogeneity of the treatment effects across patients with sufficiently different $\:X$ values). This is formalised as the maximisation of the squared difference in resulting leaf means $\:{n}_{L}\:{n}_{R}{({y}^{L}-{y}^{R})}^{2}$, where n is the number of observations in a leaf, and L and R represent the left and right leaves from a split. This procedure is performed on many random subsamples of the data, forming causal forests. Once all nuisance parameters have been estimated, the modified formula for the CATE becomes:

$$\:\widehat{\tau\:}\left(x\right)=\:\frac{\sum\:_{i=1}^{n}{\alpha\:}_{i}\left(x\right)\left\{({{W}_{i}-p\left({X}_{i}\right)\left)\right(Y}_{i}-m\left({X}_{i}\right))\right\}}{\sum\:{{\alpha\:}_{i}\left(x\right)\left\{{W}_{i}-p\left({X}_{i}\right)\right\}}^{2}}\:.$$

Honesty splitting is implemented on each tree (and a subsample of the data); the subsample is partitioned, with one part used to grow the tree and the other half used to estimate the CATE. Work by Athey and Wager shows that this algorithm leads to consistent estimators of the CATE [19]. Once CATE estimates are obtained for all observations, we can use post-estimation methods to test for heterogeneity. The CF can include many variables in a single analysis without increasing type I error. Testing for the presence of heterogeneity is done through an “omnibus test”, which uses the predicted CATEs to fit a linear predictor of the outcomes and determine the performance of our estimates, where the null hypothesis is that there is no HTE and the alternative hypothesis is that there is heterogeneity present. Output from the omnibus test includes a differential forest prediction (DFP) and associated p-value. A DFP close to 1 indicates that the CF adequately captures the underlying heterogeneity. In-built variable importance metrics are available, which utilise the frequency of which variables are chosen to be split and the stage of the tree-growing process at which they are used; see the formula in supplementary material A1. We can also observe how the CATE varies with each variable to visualise heterogeneity across baseline characteristics, for example, using scatter plots of the CATE against individual covariates. If relevant, heatmaps can be used to explore how the CATE varies across two covariates by plotting predictions of the CATE at specified quantiles of the two covariates, holding all other variables at their median.

CFs, the omnibus test and the associated variable importance, were implemented in the R grf package [27].

### Data-driven subgroups– an extension of the CF

In addition to individual-level CATEs, we sought to define data-driven subgroups of heterogeneous treatment effects to facilitate a more clinically actionable result from this model. To achieve this, we extracted root (initial) splits from each tree. Root splits are used because the first split of a causal tree maximises the difference between the ATEs in the results of two halves of the data. Individual trees are grown on different subsampled datasets; the most common first split (root) and its average represent the most likely value defining the “first-order” subgroups. The mean of the most common split was calculated to determine a data-driven threshold. Using this mean threshold, we split our trial population into two subgroups and estimate the Group ATE (GATE) in each group. The GATEs are computed using the function “average_treatment_effect” from the GRF package. The GATE estimation uses augmented inverse-propensity weighting (AIPW); see supplementary appendix A2 for the AIPW formula. AIPW involves the averaging of doubly robust scores obtained through the honesty approach [28]. We also calculate confidence intervals for the GATEs but note that this might be an underestimation of the uncertainty and, therefore, should be interpreted cautiously [29]. We hypothesise that this underestimation of uncertainty is due to the same data being used to estimate the GATEs as was used to fit the causal forest. This is a crude initial exploration of data-derived subgroups from this model.

### Implementation

Before implementing the above methods, we handled missing data in the potential effect modifiers. MissForest was used to impute the missing values [30]. Briefly, for each variable to be imputed, this approach fits a random forest on the observed part using the values available in all other variables, predicts the missing part to impute the variable at hand, and then iterates. It has a built-in stopping rule based on out-of-bag errors. Alternative missing data approaches, single mean imputation, complete-case analysis and inverse probability weighting (IPW) were carried out as sensitivity analyses. For complete-case analysis, any participants with at least one entry missing (after removal of variables with > 30% missingness) were removed from the analysis. IPWs were calculated using logistic regression, which produced probabilities of each participant having missing data and used the inverse of these probabilities as weights in the analysis. It was not possible to incorporate these weights for the hierarchical lasso.

Next, we removed highly correlated variables to attenuate issues with lasso regression and forests models, where two highly correlated variables are equally likely to be selected (for either inclusion in the final model or creating splits). Therefore, where variables had a Pearson correlation coefficient > 0.7, the variable carrying the least information (higher degree of missingness or detailing a score instead of a raw value) was excluded from the set of effect modifiers.

We also repeated the analysis without the components of the APACHE II and SOFA scores, including only the summary scores. This comparison was done in keeping with a typical approach, which assesses severity through these summary scores.

The CF [27] has several hyperparameters with defaults that can be set or tuned. Hyperparameters were set manually; full details can be found in supplementary appendix A3.

### Simulation Study

Alongside the case study analysis, we conducted a targeted simulation study to investigate the performance of our extension of the CF to identify subgroups based on thresholds using the modal initial (root) splits. The aim was to determine how often this data-driven method would identify a true subgroup effect for a continuous covariate with an underlying binary threshold value when this existed.

To do so, using a logistic model for a binary outcome, we generated 1000 trial datasets containing 1000 participants with a binary treatment indicator W (generated from a Bernoulli distribution), normally distributed continuous prognostic variable X1 and a normally distributed continuous treatment effect modifier X2 which defined a true subgroup membership using a threshold based on the observed interaction in the VANISH dataset. This meant we generated the true subgroups split (i.e. the difference in treatment response) using a threshold of 4.68 for X2. Performance was measured by the percentage of times the model correctly identified X2 as the treatment effect modifier. The mean and 95% confidence intervals of the identified thresholds are reported.

We also compared the inference obtained from using the traditional analysis approach and the hierarchical lasso looking at the treatment interaction with the continuous covariate X2, as well as the interaction with a binary subgroup covariate that dichotomised the continuous covariate at an alternative hypothesised clinically known value of 5.4 that was different to the true subgroup threshold effect. This was to mimic what could be observed in practice where these two methods can only utilise known information to categorise a continuous covariate. Full details following the ADEMP structure for planning simulations are in the Appendix [31].

We also compared the subgroup effects identified for the continuous covariate through traditional univariable interaction tests, the hierarchical lasso, and root splits of the CF where a true subgroup effect based on an underlying threshold value existed for the continuous covariate. The true threshold value for the subgroup effect was set to be different to an alternatively hypothesised known clinical value for handling that continuous covariate.

## Results

All participants from the VANISH trial were included in this analysis, and the baseline characteristics are described in Table 1. After the removal of variables with high missingness (> 30%) and high correlation (> 0.7), 49 baseline variables were available. All characteristics were reasonably well balanced between treatment arms, as expected due to randomisation.

**Table 1:** Baseline characteristics of VANISH participants

	Norepinephrine (*n* = 204)	Vasopressin (*n* = 204)	Total (*n* = 408)
Age yrs mean (SD)	63.1 (15.4)	65.1 (15.0)	64.1 (15.2)
Female n (%)	77 (37.7%)	94 (46.1%)	171 (41.9%)
Non-white ethnicity	29 (14.2%)	31 (15.2%)	60 (14.7%)
BMI Mean (SD)	26.8 (6.75)	27.4 (7.84)	27.1 (7.32)
Hours shock to randomisation mean (SD)	3.79 (6.63)	3.48 (2.59)	3.63 (5.03)
Surgical admission n (%)	35 (17.2%)	38 (18.6%)	73 (17.9%)
**Source of infection n (%)**			
Lung	83 (40.7%)	82 (40.2%)	165 (40.4%)
Abdomen	47 (23.0%)	45 (22.1%)	92 (22.5%)
Tissue	9 (4.4%)	10 (4.9%)	19 (4.7%)
Other	61 (29.9%)	62 (30.4%)	123 (30.1%)
**Physiological Measurements**			
Etomidate used n (%)	6 (2.9%)	5 (2.5%)	11 (2.7%)
Previous vasopressor use n (%)	168 (82.4%)	180 (88.2%)	348 (85.3%)
IV fluids in last 4 h mls mean (SD)	1550 (1360)	1500 (1180)	1520 (1270)
Synthetic starch use n (%)	13 (6.4%)	12 (5.9%)	25 (6.1%)
Lactate mean mmol/L(SD)	3.12 (2.52)	3.31 (2.90)	3.22 (2.72)
Mechanical ventilation n (%)	123 (60.3%)	113 (55.4%)	236 (57.8%)
Renal replacement therapy n (%)	5 (2.5%)	6 (2.9%)	11 (2.7%)
Bilirubin mg/dL mean (SD)	1.65 (2.68)	1.66 (2.31)	1.66 (2.50)
Platelet x10^3^ /µl mean (SD)	224 (148)	211 (134)	218 (141)
**Comorbidities**			
Ischemic heart disease n (%)	31 (15.2%)	31 (15.2%)	62 (15.2%)
Chronic kidney failure n (%)	10 (4.9%)	17 (8.3%)	27 (6.6%)
Cancer n (%)	22 (10.8%)	24 (11.8%)	46 (11.3%)
Immunocompromised n (%)	15 (7.4%)	13 (6.4%)	28 (6.9%)
Kidney failure at baseline n (%)	47 (23.0%)	38 (18.6%)	85 (20.8%)
Diabetic n (%)	51 (25.0%)	39 (19.1%)	90 (22.1%)
**SOFA scores**			
**Total score**	6.82 (2.98)	6.80 (3.03)	6.81 (3.00)
Coagulation mean (SD)	0.575 (0.910)	0.644 (0.936)	0.610 (0.923)
Cardiovascular mean (SD)	2.99 (1.42)	3.00 (1.38)	2.99 (1.40)
**APACHE II scores**			
**Total score**	24.3 (7.88)	24.8 (7.95)	24.5 (7.91)
Min. temperature ^o^C Mean (SD)	36.1 (1.20)	36.0 (1.21)	36.1 (1.20)
Max. temperature ^o^C Mean (SD)	37.7 (1.30)	37.7 (1.24)	37.7 (1.27)
Min. mean arterial pressure mmHg Mean (SD)	57.1 (10.5)	56.5 (9.54)	56.8 (10.0)
Max. mean arterial pressure mmHg Mean (SD)	89.9 (17.0)	89.3 (17.4)	89.6 (17.2)
Min heart rate bpm Mean (SD)	85.8 (19.0)	85.0 (19.5)	85.4 (19.2)
Max. heart rate bpm Mean (SD)	120 (25.7)	122 (26.0)	121 (25.8)
Min. respiratory rate bpm Mean (SD)	16.7 (5.41)	16.3 (5.03)	16.5 (5.22)
Max. respiratory rate bpm Mean (SD)	31.5 (9.29)	30.5 (9.25)	31.0 (9.27)
Min PaO2 kPa Mean (SD)	9.94 (3.54)	11.0 (6.84)	10.5 (5.46)
PaCO2 at min PaO2 kPa Mean (SD)	5.32 (1.80)	5.40 (2.21)	5.36 (2.02)
Max. FiO2 Mean (SD)	0.679 (0.259)	0.702 (0.259)	0.690 (0.259)
PaO2 at max FiO2 kPa Mean (SD)	15.8 (10.0)	16.6 (10.0)	16.2 (10.0)
PaCO2 at max FiO2 kPa Mean (SD)	5.65 (1.97)	5.69 (2.37)	5.67 (2.18)
Min pH Mean (SD)	7.24 (0.134)	7.25 (0.144)	7.25 (0.139)
Max pH Mean (SD)	7.37 (0.107)	7.38 (0.0967)	7.37 (0.102)
Max Na mmol/L Mean (SD)	137 (6.03)	138 (5.66)	138 (5.85)
Max K mmol/L Mean (SD)	4.66 (1.03)	4.50 (0.822)	4.58 (0.937)
Max Creatinine µmol/L Mean (SD)	187 (189)	172 (129)	179 (162)
Acute Renal Failure n (%)	111 (54.4%)	103 (50.5%)	214 (52.5%)
Max haemoglobin g/dL % Mean (SD)	12.1 (2.48)	12.0 (2.53)	12.0 (2.50)
Max white blood cell count x10^3^ /µl Mean (SD)	18.7 (24.5)	18.2 (26.4)	18.4 (25.4)
Lowest Glasgow Coma Score Median (IQR)	14 (10, 15)	14 (11, 15)	12.1 (3.98)
**Outcome measures**			
No kidney failure n (%)	107 (52.5%)	117 (57.4%)	224 (54.9%)
Survived to 28 days n (%)	148 (72.5%)	141 (69.1%)	289 (70.8%)
Renal failure-free days n (%)	19.7 (11.1)	19.2 (11.5)	19.5 (11.3)

This table reports summary statistics for the entire randomised trial population; the data have already been imputed using the MissForest package. Table only reports variables included in the analysis; variables with high missingness and correlation are excluded

### Univariable regression

**Fig. 1:**
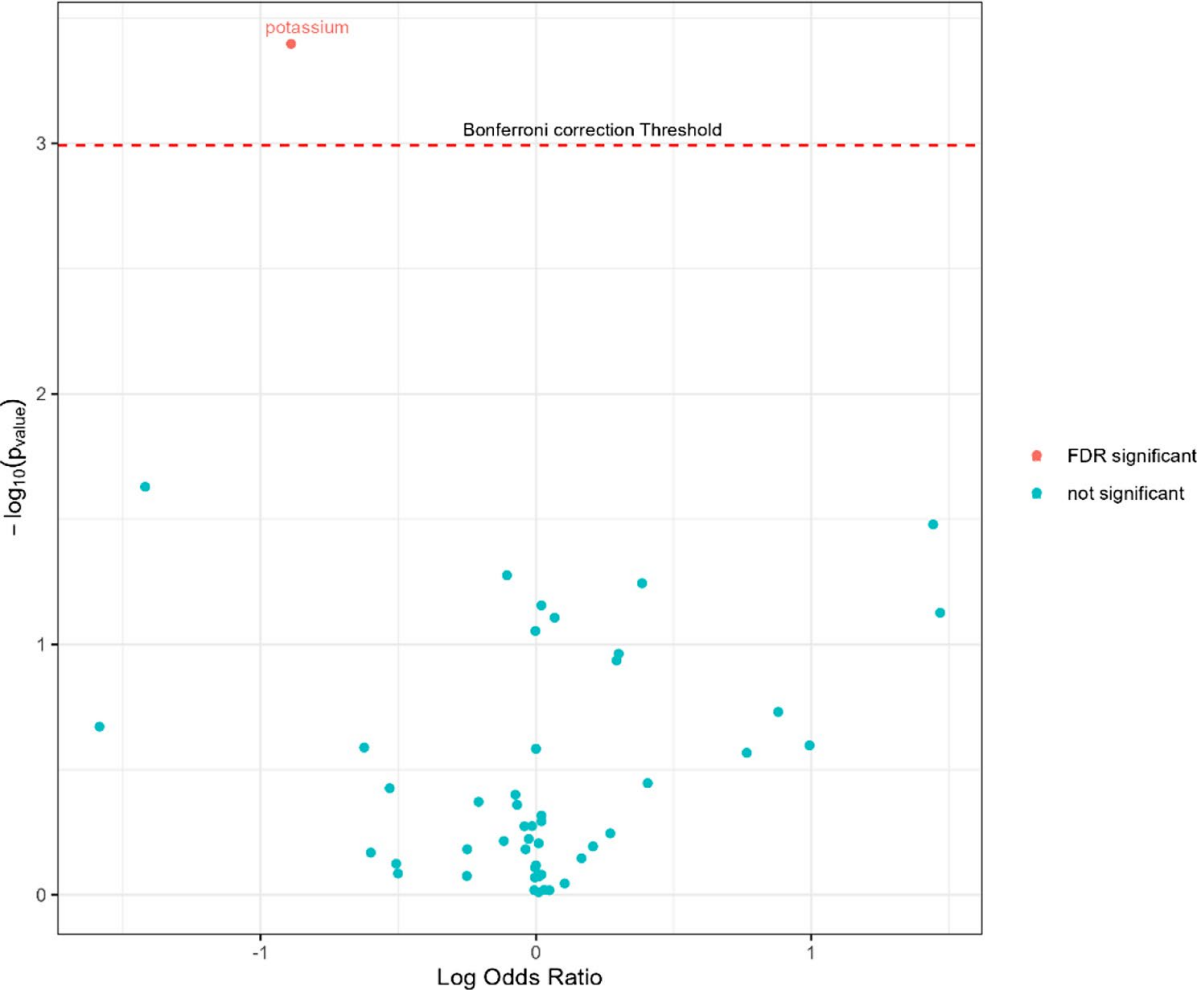
Volcano plot of interaction terms from the univariable approach. Y axis is negative log base 10 to show so that smaller p-values place higher on the figure. Each point shows the log odds ratio for the interaction term between a patient covariate and the treatment. Red points are significant with a False Discovery Rate (FDR) below 0.05, points above the red dotted line are Bonferroni significant to 0.05.

The Bonferroni significance threshold for the univariable analysis was *p* = 0.00102 (0.05/49). Figure 1 displays the interaction coefficients resulting from the univariable analysis in a volcano plot. The interaction between maximum serum potassium in the 24 h before ICU admission (APACHE potassium) was OR 0.435 (95%CI [0.270, 0.683]. *p* = 0.0004), indicating that increased levels of potassium in the blood were associated with a poorer chance of survival on the treatment. Ischemic heart disease was also associated with a poorer chance of survival on the treatment (OR: 0.600, 95%CI [0.379, 0.923], *p* = 0.024) and having cancer was associated with an increased chance of survival on the treatment (OR: 1.58, 95%CI [1.04, 2.44]. *p* = 0.033); however, these two findings were not significant after multiple testing corrections.

### Hierarchical lasso

**Fig. 2:**
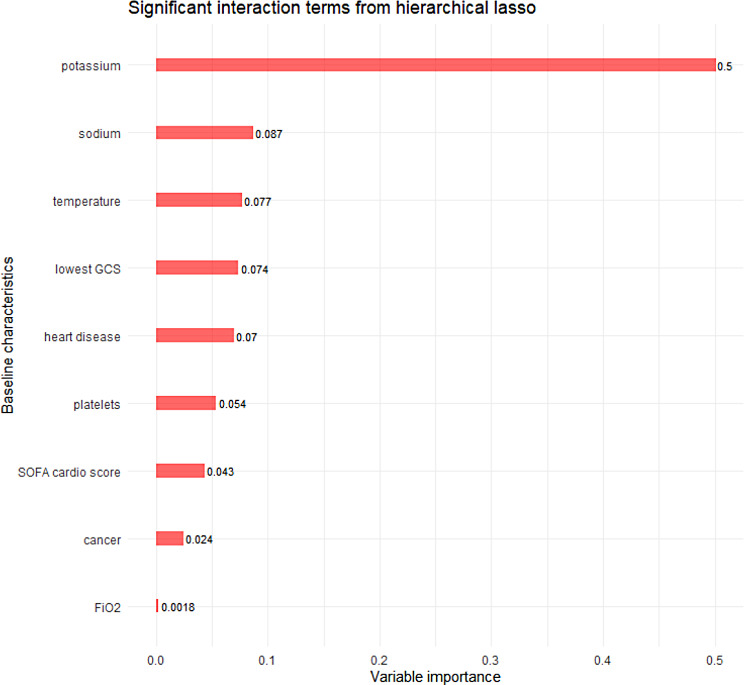
Selected interaction terms from the hierarchical lasso. Magnitude of the treatment-covariate interactions kept by the hierarchical logistic lasso model. Log odds ratios are displayed Since all covariates were scaled, the magnitude of the terms represents the variable importance. Confidence values were not calculated for these estimates

The hierarchical lasso model kept nine interactions and their corresponding main effects, and the magnitude of these interaction effects is displayed in Fig. 2. The optimal penalty term lambda was 0.0049, and the cross-validation error was 0.494. The nine interaction terms retained in the model were cancer comorbidity, ischemic heart disease, Glasgow coma score, maximum FiO_2_, maximum potassium, maximum sodium, minimum temperature, platelet count, and cardiovascular SOFA score. Potassium was associated with a decreased chance of being alive at 28 days (standardised OR: 0.604, 95% CI (0.259, 0.701)). Since all baseline characteristics were centred and scaled, the magnitude of the log odds is interpretable as a measure of variable importance.

### Causal forest

**Fig. 3:**
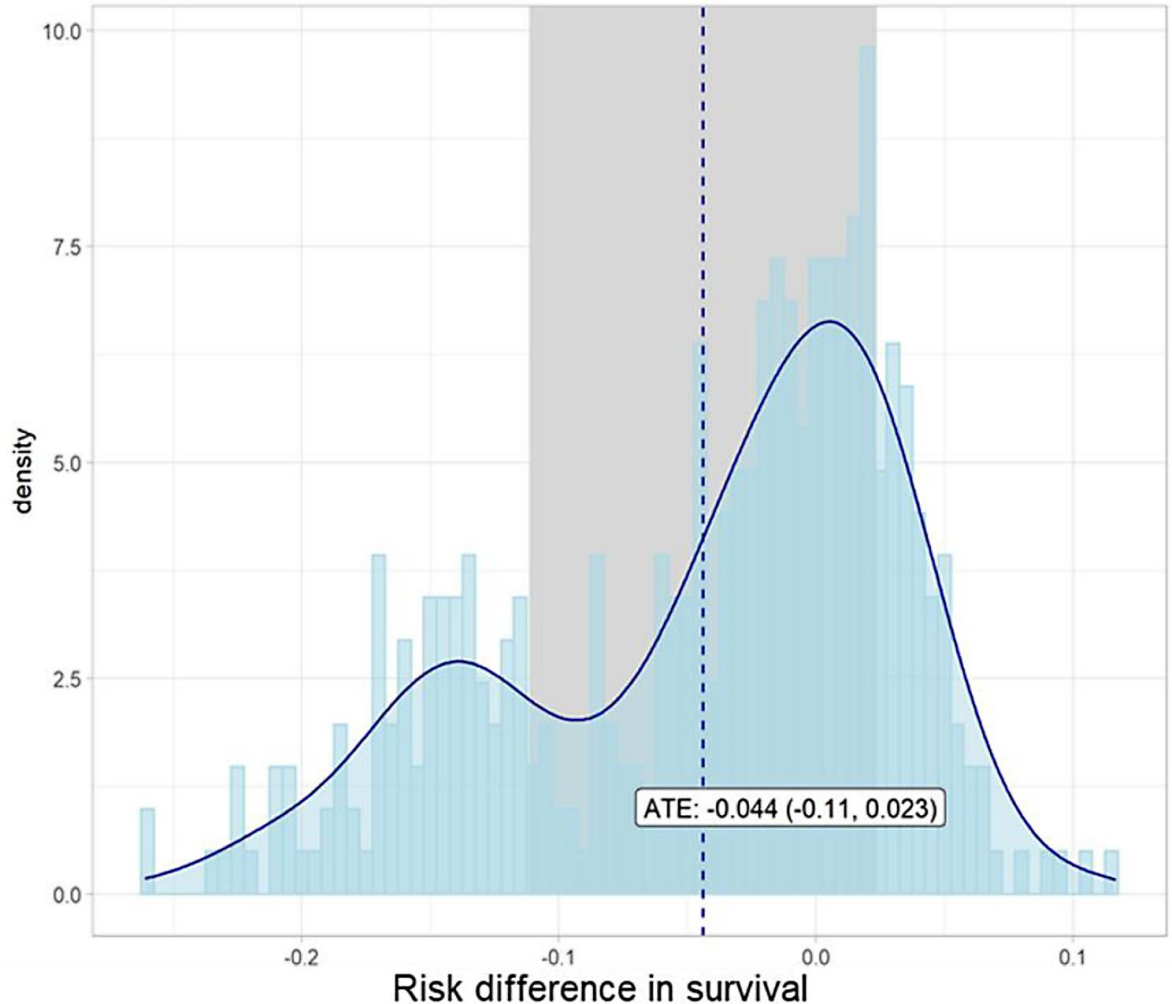
Estimated distribution of the CATE by causal forest. The dashed line represents the ATE of the whole population and the grey area represents the 95% confidence interval for this estimate. In blue, is a histogram and density line representing the spread of the individual CATEs

The causal forest found weak evidence of HTEs through the omnibus test, which gave a DFP of 0.732 (*p* = 0.124). The variation in the conditional average treatment effect and its associated confidence interval can be seen in Fig. 3, which shows that the distribution of the CATE in this trial population is approximately bimodal. This bimodal distribution indicates that partitioning this population may result in subpopulations with significantly different ATEs.

Examination of the most common root splits gives the same ordering of variable importance as the inbuilt function (see supplementary appendix C). Examining the most popular root split, the most important variable, enabled the partitioning of the sample population into two subgroups with different ATEs.

**Fig. 4:**
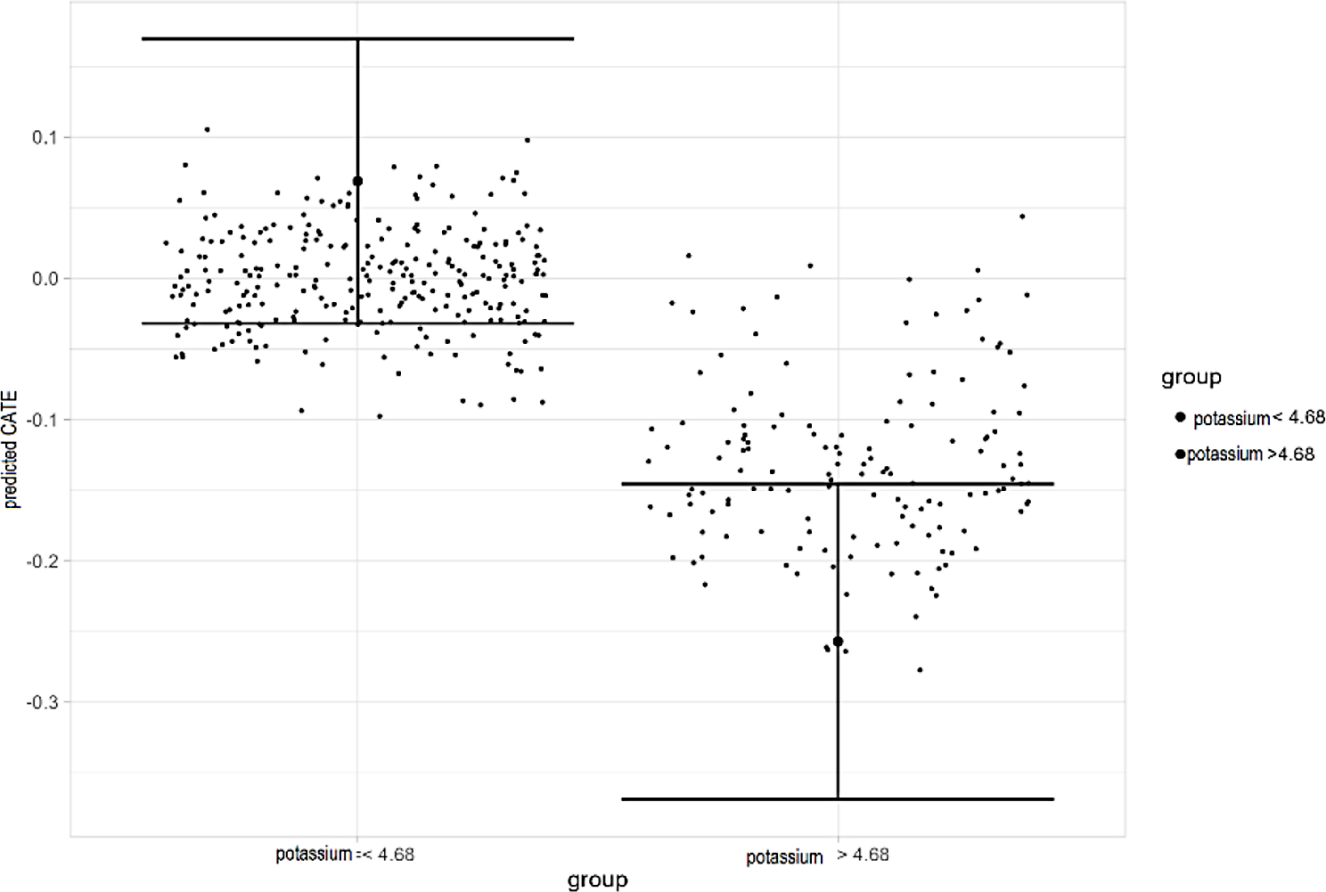
Data-driven subgroups from the VANISH study population. ATEs for the subgroups results from the most common root splits. ATEs and SEs are estimated using AIPW, confidence intervals are not for causal interpretation, they are for hypothesis generation only

In this model, the most common split was on serum potassium, with a mean value of 4.68 mmol/L. This value was used to partition the patient population into low and high potassium (below and above the 4.68 mmol/L threshold). The resulting subgroup GATEs and their confidence intervals are shown in Fig. 4. The subgroup GATEs (risk difference in 28-day mortality) for the low potassium and high potassium groups were 0.069 (95%CI [-0.032, 0.169]) and − 0.257 (95%CI [-0.368, -0.146]) respectively. We note that the confidence intervals of the GATEs in Fig. 4 appear skewed, and we hypothesise that this is due to the use of the AIPW in GATE calculation. Treatment allocation was no longer balanced between subsets, leading to skew in the calculation of GATEs. Common splits, their thresholds and resulting GATEs are presented in supplementary appendix D.

### Sensitivity analyses

#### Comparing summary scores to individual components

For the primary analysis, the components of the summary scores were included rather than the summary scores. The opposite situation was investigated in a sensitivity analysis, and summary scores were included rather than components, see supplementary appendix B1. For the causal forest, there was no strong signal of heterogeneity (differential forest prediction was − 7.73, *p* = 0.99). Omnibus test results for all summary scores analysis are available in supplementary appendix B1. Here, we can see that the inclusion of components of summary scores has helped to discover variations in treatment effect that would have been missed if only summary scores had been considered.

**Sensitivity analysis to missing data strategy**: Several approaches to handling missing data were trialled in this exploration: mean imputation, compete-case analysis, and IPW. For IPW and complete cases, 255 participants were included in the analysis. The latter two approaches significantly decreased the available sample size. Mean imputation yielded results similar to those of the primary analysis, with the same conclusions. Results using alternative missing data methods are outlined in the supplementary appendix B2.

### Simulation Study

**Fig. 5:**
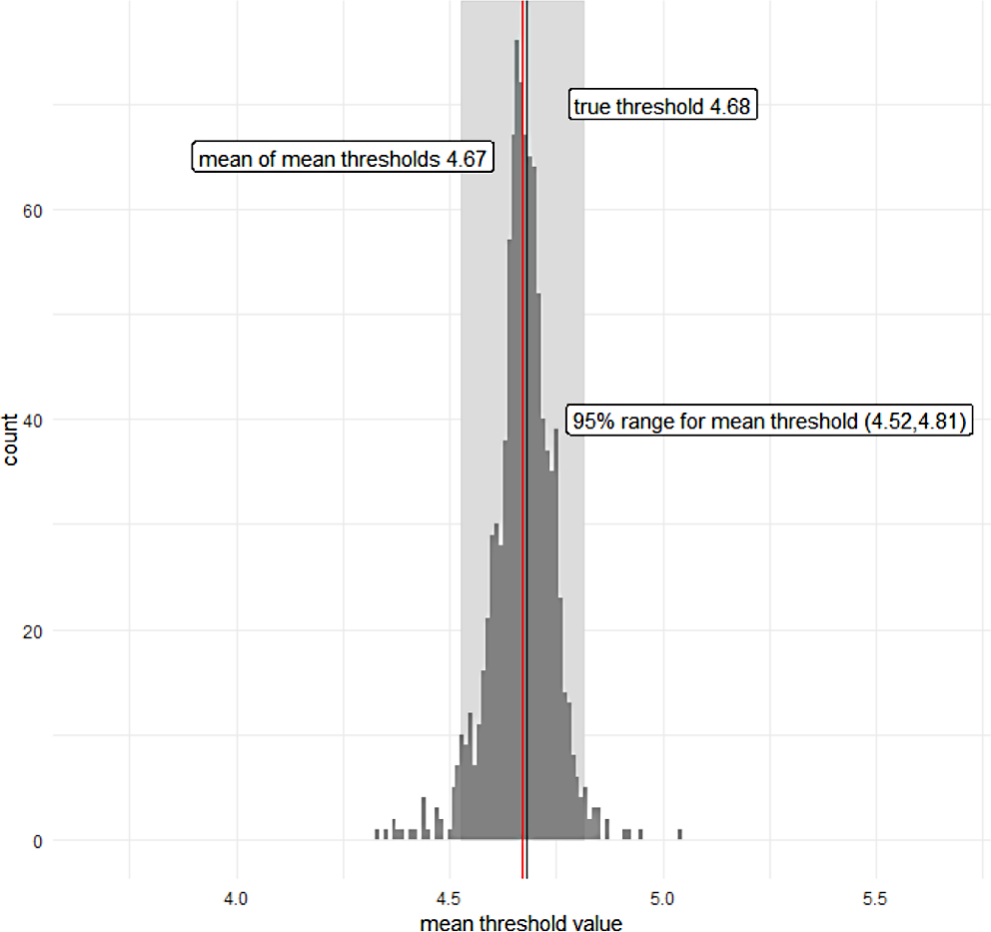
Histogram of the mean root splits obtained from the causal forest, including the mean of mean thresholds (red) and the true threshold (black), and 95% range

The simulation performed to study the performance of our extension to the CF to identify a subgroup resulted in correctly identifying X2 as a treatment effect modifier 100% of the time across all causal forest simulations since X2 was the modal root split. The mean of the mean thresholds across simulations was 4.671 (SD: 0.0725) (MSE from true threshold = 0.00534). Figure 5 shows the mean thresholds obtained by the causal forest. The 95% range of values was from 4.52 to 4.81.

In the traditional univariable analysis, when testing for interactions between the continuous covariate X2 and the treatment, 99.5% of simulations detected a significant interaction with the continuous covariate (*p* < 0.05). However, as this method is not data-driven, this method was not able to reveal the true subgroup threshold effect of interest (0% of simulations). When using the hypothesised known clinical threshold to dichotomise the continuous covariate that did not match the true subgroup threshold and testing the interaction between the clinically dichotomised X2 and treatment, 83.7% of simulations detected a significant interaction.

In the hierarchical lasso with the continuous X2 covariate, all simulated models kept the interaction between the continuous covariate and the treatment. 99.1% of simulations detected a significant interaction between the continuous covariate and the treatment (*p* < 0.05). However, similarly to the univariable analysis, this method could not reveal the true subgroup threshold effect of interest (0% of simulations). For the dichotomised X2 covariate based on the known clinical threshold that did not match the true subgroup threshold, 99.7% of simulations kept the interaction term between the treatment and categorical variable. Of these simulations, 99.9% of models detected a significant interaction between the categorical covariate and the treatment.

## Discussion

This secondary analysis of the VANISH trial demonstrated evidence of treatment effect heterogeneity with variation in serum potassium levels. Higher potassium levels were associated with poorer treatment response. High potassium levels, known as hyperkalaemia, can occur in the later stages of renal dysfunction [32]. This relates to the idea that patients with more severe or later-stage sepsis who have already developed kidney injury are unlikely to benefit from early vasopressin, an idea which has been postulated previously [33].

The univariable, hierarchical lasso, and causal forest all identified serum potassium levels as an important driver of HTEs in the VANISH trial population. Although the regression-based techniques were able to detect the negative relationship between serum potassium and treatment response, the causal forest went a step further and additionally identified two subgroups within the study population, which had distinct treatment effects. The cut-off that defined these subgroups was within the normal range for potassium (3.5–5.5 mmol/L) [34] and might not have been considered as a potential partition value without the implementation of CFs.

This re-analysis highlights a finding for further research that would have otherwise been missed. Hyperkalaemia is associated with more severe, late-stage sepsis, but in a previous reanalysis by baseline risk of death determined by the full APACHE II score [35], there was no significant variation in HTE between the highest and lowest quartiles of baseline APACHE II scores. Patients with similar total APACHE II scores might have different aetiologies and, hence, different responses to treatment. CFs are better equipped to avoid an increased type I error rate [36] which occurs when using classical univariable methods which carry out many individual tests.

A CF analysis was previously conducted for the 65-trial, which evaluated the effect of a permissive hypotension strategy versus usual care on 90-day mortality for critically ill patients aged 65 or older with vasodilatory hypotension. The CF re-analysis of the 65-trial [20], was compared with the earlier pre-specified subgroup analysis, which used classical methods [37]. The CF re-analysis similarly added to the pre-specified subgroup analysis that had already been done and proposed further hypotheses about treatment effect heterogeneity that had not been previously considered.

All analysis described in this study is exploratory. From the baseline data collected in the trial, we post-hoc aimed to identify potential interactions to be investigated further. This aim is different from that of a pre-planned subgroup analysis, which tests specific hypotheses [13]. With this, it is acknowledged that these findings might be underpowered as subgroups are smaller and will require cautious interpretation and validation in other cohorts.

Our targeted simulation study showed that the causal forest combined with root splitting could discover subgroup effects for continuous covariates defined by underlying subclinical thresholds. When using a typical regression interaction model or even the data-adaptive hierarchical lasso, we can identify a signal of heterogeneity along a continuous covariate. Still, we cannot identify a threshold for splitting into subgroups of HTEs. Using existing clinical thresholds to define subgroups may not be optimal and make subgroup effects more difficult to detect.

The root split values come from different subsampled datasets, but we have not shown that using this mean root split to define subgroups can yield valid uncertainty estimates. We do not recommend that any data-derived subgroups (whether defined by these thresholds or otherwise) inform practice; we see them instead as subgroup hypothesis generation to be tested and validated in further research. Although our simulation study showed good recovery of subgroups through mean root splits on a continuous variable, further methodological work is required to construct valid confidence intervals for these estimates. We are now conducting more extensive simulation studies as part of further work.

Possible refinements to this simple subgrouping might include using the second most important variable to create multiple or more detailed subgroups. Alternatively, finding multiple thresholds on a single variable may be of interest, for instance, to define positive, negative, and neutral responders to an intervention. Classification Analysis (CLAN) is a technique to compare the average characteristics of the least and most affected groups in a study [23]. However, this method does not group the entire study population and does not necessarily yield clinically actionable thresholds.

The EMA guidelines for subgroup investigations in confirmatory clinical trials generally note that biological plausibility and replicating subgroup findings in other trials increase the credibility of subgroup findings [38]. Such steps can make findings more clinically actionable. Newer methods for estimating subgroup effects may be used to pre-plan and power subgroup analysis in trials, or external validation could be sought in existing datasets to further increase the credibility of findings from such methods.

Understanding how uncertain subgroup effects are is paramount for these methods to contribute fully to the world of personalised medicine [39]. Human-in-the-loop research allows for algorithms and clinicians to collaborate, where clinicians can oversee algorithmic decisions and override algorithms when the uncertainty is high [40]. Extensions of CATE estimation for the future can include modelling complex outcomes, such as competing events and sequential decisions.

The co-primary outcome of the VANISH study was kidney failure-free days during the 28 days post-randomisation. To analyse this outcome appropriately, we would need to consider the competing risk of death, and CF methods are currently unable to account for this risk. Further research is required in order to develop this methodology for use in this setting.

We acknowledge that the VANISH trial population was small, and many applications of machine learning methods are done in larger datasets. However, the results from the CF were consistent with those from the univariable and Lasso models, which gives confidence in the results seen in this paper. Further work is needed to understand where limits to sample size impact the interpretation of results, what adequate sample size is when implementing CFs, and the need for external validation of the findings before using this for clinical decisions.

Several methods for handling missing data were compared in this study. Best practice methods are yet to be established, but the present study found little difference between mean imputation and non-parametric single imputation. This may have been the case as the variables are not confounders but baseline variables in a randomised trial. Other potential methods included IPW and complete-case analysis. In this study, these methods significantly reduced the size of the dataset and made comparisons between methods difficult. It would, therefore, be interesting to apply these methods to a larger dataset and see how they differ from imputation approaches. Multiple imputation is another candidate method to be explored as this is a common, favoured method in classical analysis approaches but needs to be incorporated into the workflow of machine learning applications [41].

## Conclusion

This study has demonstrated the potential of using CFs to identify HTEs in a post-hoc context, generating hypotheses which can be investigated further in other studies. CFs can handle large numbers of variables, allowing investigation of heterogeneity along individual components of disease severity rather than just overall scores, maximising the use of available information. CF-generated cut-offs are a novel concept that has added value over other methods or a priori chosen thresholds to find thresholds that define heterogeneity that clinicians or other researchers may not have previously thought of.

## Electronic supplementary material

Below is the link to the electronic supplementary material.

Supplementary Material 1

## Data Availability

The datasets analysed during the current study are not publicly available due to lack of consent for data to be made public, which was not collected in the original trial but are available from the corresponding author on reasonable request.

## Abbreviations

HTE: Heterogeneous treatment effect
ATE: Average treatment effect
AIPW: Augmented inverse–propensity weighting
CATE: Conditional average treatment effect
GATE: Group average treatment effect
ICU: Intensive care unit
CF: Causal forest
SOFA: Sequential organ failure assessment
APACHE II: Acute physiology and chronic health evaluation II
RCT: Randomised controlled trial
