# Supplementary material for: Application of causal forests to randomised controlled trial data to identify heterogeneous treatment effects: a case study
Source: BMC Med Res Methodol. 2025 Feb 22;25:50. doi: 10.1186/s12874-025-02489-2 (PMC11846376; doi:10.1186/s12874-025-02489-2)
Supplement: Supplementary file 1 — Supplementary Material 1 [file 12874_2025_2489_MOESM1_ESM.docx]

**Supplementary Appendix**

1. Supplementary methods material
   1. Formula for variable importance in the causal forest

$$imp\left( x_{j} \right)= \frac{\sum_{k=1}^{4} \left[ \frac{\sum_{all trees} number of k depth splits on x_{j}}{\sum_{all trees} total number of depth k splits} \right]k^{-2}}{\sum_{k=1}^{4} k^{-2}}$$

The importance of a variable $x_{j}$ in the causal forest is given by the formula above. For the first 4 ($k$) levels of splits in every tree (the depth), the number of splits on the variable $x_{j}$ are counted and the number of splits on any variables are counted. A maximum depth of 4 is chosen for computational efficiency.

- 1. formula for Augmented inverse propensity weighting for calculating ATEs

$$\hat{\tau}^{AIPW}= \frac{1}{n}\sum_{i=1}^{n} \hat{\mu}^{-i}\left( X_{i},1 \right)- \hat{\mu}^{-i}\left( X_{i},0 \right)+ \frac{W_{i}}{\hat{e}^{-i}\left( X_{i} \right)}\left( Y_{i}-\hat{\mu}^{-i}\left( X_{i},1 \right) \right)- \frac{1-W_{i}}{1- \hat{e}^{-i}\left( X_{i} \right)}\left( Y_{i}-\hat{\mu}^{-i}\left( X_{i},0 \right) \right)$$

Here, $\hat{\mu}^{-i}$ indicates the estimate was generated on subsamples not containing the ith observation.

- 1. Choices of tuning parameters for the causal forest with justifications

Due to the small size of the dataset used in this project, most hyperparameters were set manually. The parameter “honesty” was set as TRUE, ensuring sample splitting was used to separate split decisions and CATE estimations. The “honesty.fraction”, the proportion of the data used for determining splits, was set to 0.7 so that more data could be used for deciding on placements of splits. Estimation of sample trees was skipped if they contained empty leaves by setting “honesty.prune.leaves” to false, this helps to improve performance with smaller datasets. The maximum imbalance of a split “alpha” was set as 0.1, allowing a 10% imbalance in splits. The number of variables considered at each split, “mtry”, was set to the total number of variables included in the model. The “imbalance.penalty” was set to 0.01, allowing a small penalty for imbalanced splits. The “min.node.size” was tuned for each forest, and 2000 trees were used for tuning. The number of trees per forest was set to 50,000 rather than the default of 5000 due to the small size of the input data. 10-fold cross-validation was employed in the forest by setting the clusters as folds of the data. This helped to maximise the use of the small dataset available.

1. 28-day mortality outcome results with different missing data handling methods
   1. All uncorrelated variables (as per primary analysis)

These graphs follow the same format as the causal forest plot in the main article. The dashed line and grey bar represent the average treatment effect (ATE) and it’s 95% confidence interval. The Blue bars represent individual treatment effects, and the dark blue line shows the distribution of the conditional average treatment effect (CATE). The CATE and ATE are the risk difference of being alive at day 28, the primary outcome in this analysis. In the following graphs, the risk differences are along the x-axis labelled tau hat.

For the mean imputation and missForest methods, 408 participants included in the analysis. For the complete case and IPW methods, 255 participants were included in the analysis.


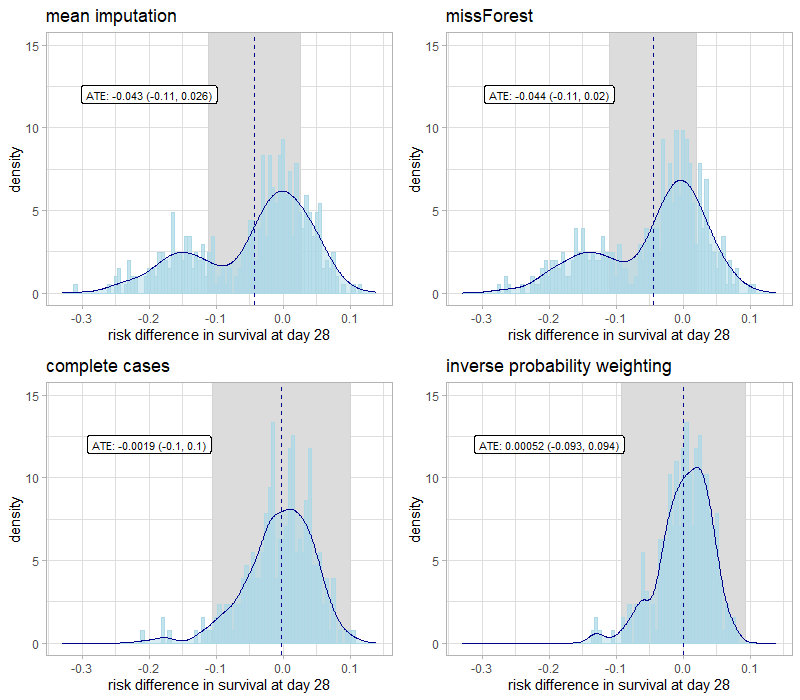


Figure a: CATE distributions for uncorrelated variables

Table a: omnibus test results for alternative missing data handling strategies

| missing data strategy | differential forest prediction | p-value |
| --- | --- | --- |
| missForest | 0.482 | 0.187 |
| mean imputation | 0.649 | 0.138 |
| complete case | -1.05 | 0.82 |
| Inverse probability weighting | -2.89 | 0.999 |

B2: Summary scores and baseline variables only (excluding components of the summary scores)


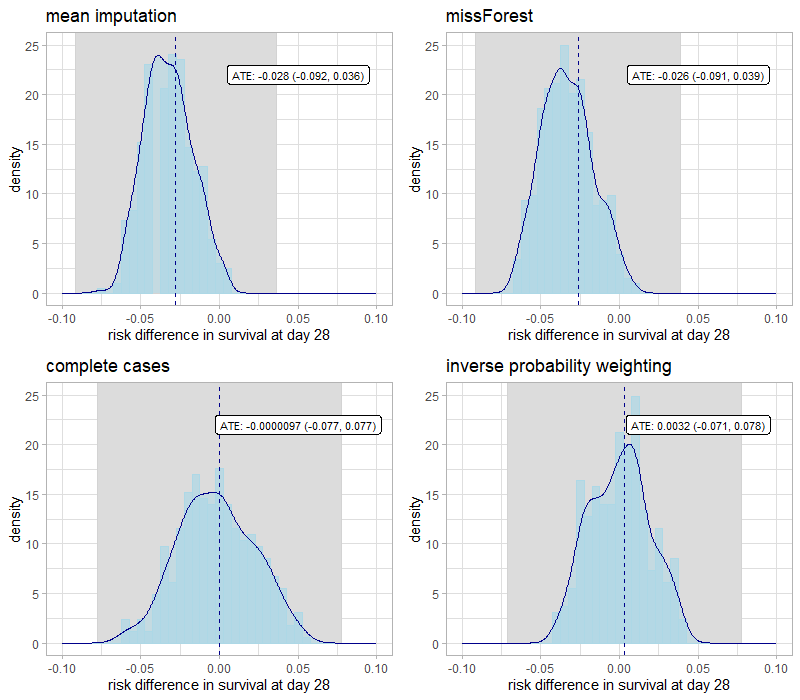


Figure b: CATE distributions for summary scores and baseline variables only

Table b: omnibus test results for analysis using summary scores and baseline variables only

| missing data strategy | differential forest prediction | p-value |
| --- | --- | --- |
| missForest | -7.73 | 0.999 |
| mean imputation | -6.65 | 0.999 |
| complete case | -4.05 | 0.999 |
| Inverse probability weighting | -6.64 | 0.999 |

1. Hierarchical lasso
   1. Variable importance plot for causal forest with all uncorrelated predictors with <30% missing and missing values imputed using missForest (as per primary analysis)

WBC stands for white blood cell count


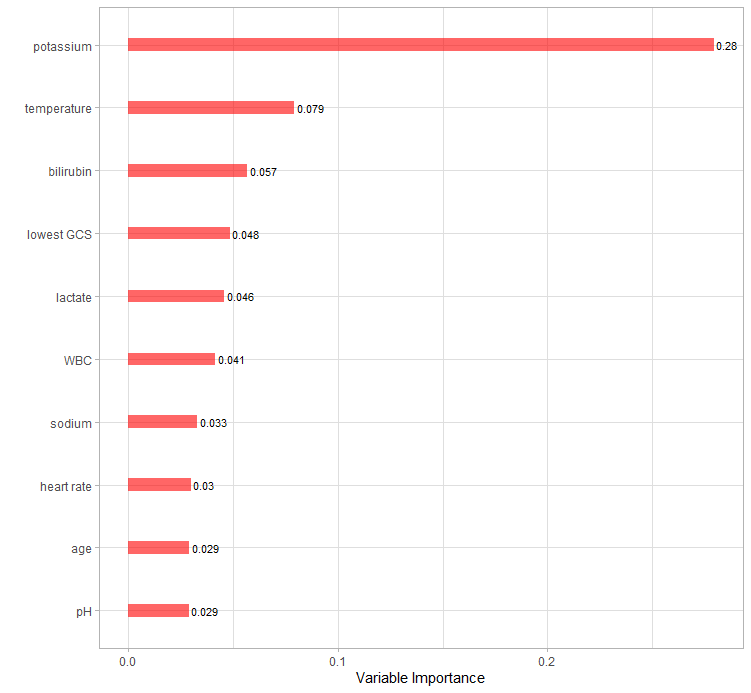


Figure c: variable importance for the causal forest primary analysis

C2. Post-selection confidence intervals for primary analysis with hierarchical lasso


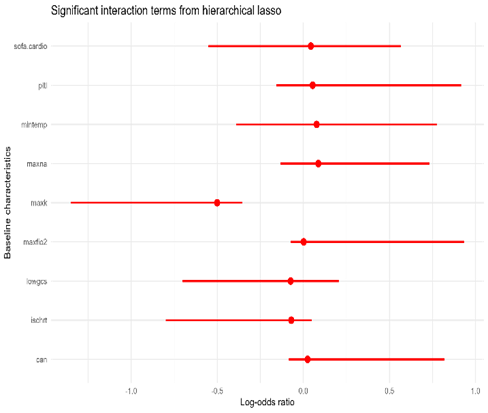


Figure d: post-selection confidence intervals for heirarchical lasso interaction terms

D – most common splits and subgroups for the other missing data strategies

Table c: root split threshold for alternative missing data handling strategies

| missing data strategy | most common root split variable | threshold | below threshold GATE | above threshold GATE |
| --- | --- | --- | --- | --- |
| missForest | Potassium (mmol/L) | 4.68 | 0.069 (-0.032, 0.169) | -0.257 (-0.368, -0.146) |
| mean imputation | Potassium (mmol/L) | 4.64 | 0.075 (-0.032, 0.182) | -0.267 (-0.386, -0.147) |
| complete case | Potassium (mmol/L) | 4.72 | 0.131 (-0.003, 0.266) | -0.279 (-0.496, -0.063) |
| Inverse probability weighting | heart rate (bpm) | 79 | -0.167 (-0.348, 0.014) | 0.107 (0.003, 0.209) |

E – example of visualising point estimates of CATEs via scatter graph


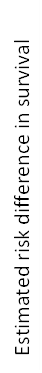
*
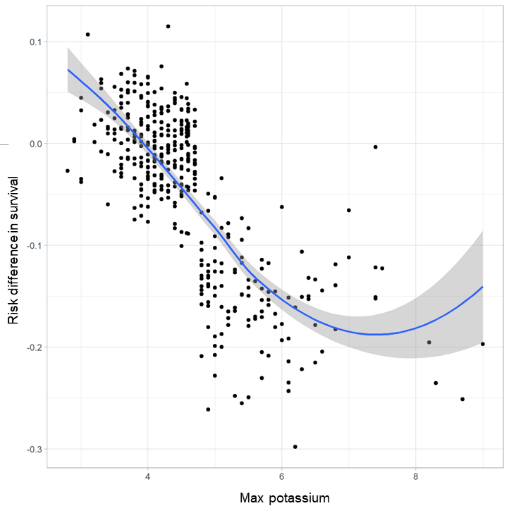
*

Figure e: CATE estimates ordered by their maximum potassium value. Estimates are out-of-bag predictions from the causal forest.

**D – ADEMP simulation plan**

**Aim:**

Check the credibility of the root splits method by assessing the number of times this method identified the true subgroup effect and the associated generated threshold value. Inference was compared against subgroup effects that would be identified through i) the classical approach of conducting univariable interaction tests and (ii) data-adaptive methods utilising the hierarchical lasso based on known information for subgrouping the continuous covariate only.

**Data Generating Mechanism:**

We will simulate the data based on the VANISH dataset (x1000). Each dataset contained 1000 participants

$$Y \sim X_{1} + W*X_{2} + \varepsilon$$

$$logit(y_{i}) = \alpha+ \beta_{1}x_{1,i} + I(x_{2,i}<4.58)*\beta_{2}w_{i}x_{2,i} + I(x_{2,i}\geq4.68)\beta_{3}w_{i}x_{2,1}$$

W~ Bernoulli(0.5)

$\alpha$ = -0.969, log odds of VANISH control mortality rate of 0.275

$\beta_{1}$ = 0.8, the prognostic effect of X1

$X_{1} \sim N(0,1)$ – prognostic variable

$\beta_{2} = -0.581$ smaller effect

$X_{2} \sim N(4.58, 0.95)$ – based on potassium levels observed in VANISH

$\beta_{3}=1.27$ – larger subgroup effect

$\varepsilon\sim N(0,1)$ error

**Estimand:**

In the causal forest, the target is the threshold defined by the root-splitting method. The target of the analysis in the logistic regression and hierarchical lasso cases is the interaction between X_2 and W where X_2 was treated as a continuous covariate or a binary covariate using a hypothesised known clinical threshold to dichotomise the continuous covariate that did not match the true subgroup threshold.

**Method:**

The causal forest was applied to find the threshold for the subgroup effect using the root-splitting method described in the main text.

Univariable interaction tests & hierarchical lasso were applied using logistic models on the continuous covariates and choosing an arbitrary boundary based on clinical guidelines (using 5.4 threshold).

**Performance metrics:**

Mean and 95% range of thresholds chosen by the CF. In all models, the percentage of runs where the model correctly identifies the subgroup effect.
